# [Retracted] Long non‑coding RNA TMCC1‑AS1 predicts poor prognosis and accelerates epithelial‑mesenchymal transition in liver cancer

**DOI:** 10.3892/ol.2024.14785

**Published:** 2024-11-04

**Authors:** Cheng Chen, Na Su, Guiying Li, Yanfeng Shen, Xiaoting Duan

Oncol Lett 22: 773, 2021; DOI: 10.3892/ol.2021.13034

Following the publication of this paper, it was drawn to the Editor's attention by a concerned reader that certain of the colony formation assay data shown in Fig. 3C on p. 5 had already appeared in a number of previously published articles written by different authors at different research institutes, a couple of which have subsequently been retracted. Owing to the fact that the contentious data in the above article had already been published prior to its submission to *Oncology Letters*, the Editor has decided that this paper should be retracted from the Journal. The authors were asked for an explanation to account for these concerns, but the Editorial Office did not receive a reply. The Editor apologizes to the readership for any inconvenience caused.

